# The pharmacological and non-pharmacological treatment of attention deficit hyperactivity disorder in children and adolescents: protocol for a systematic review and network meta-analysis of randomized controlled trials

**DOI:** 10.1186/s13643-015-0005-7

**Published:** 2015-02-27

**Authors:** Ferrán Catalá-López, Brian Hutton, Amparo Núñez-Beltrán, Alain D Mayhew, Matthew J Page, Manuel Ridao, Aurelio Tobías, Miguel A Catalá, Rafael Tabarés-Seisdedos, David Moher

**Affiliations:** Fundación Instituto de Investigación en Servicios de Salud, Valencia, Spain; Clinical Epidemiology Program, Ottawa Hospital Research Institute (OHRI), Ottawa, ON Canada; Department of Epidemiology and Community Medicine, Faculty of Medicine, University of Ottawa, Ottawa, ON Canada; Centro de Atención Integral a Drogodependientes (CAID) Norte, Regional Health Council, Madrid, Spain; Australasian Cochrane Centre, School of Public Health and Preventive Medicine, Monash University, Melbourne, Australia; Instituto Aragonés de Ciencias de la Salud (IACS), Red de Investigación en Servicios de Salud en Enfermedades Crónicas (REDISSEC), Zaragoza, Spain; FISABIO-Salud Pública, Valencia, Spain; Spanish Council for Scientific Research (CSIC), Barcelona, Spain; University of Valencia, Valencia, Spain; University of Valencia/CIBERSAM, and Instituto de Investigación Sanitaria de Valencia (INCLIVA), Valencia, Spain

**Keywords:** Attention deficit hyperactivity disorder, ADHD, Children, Treatment, Meta-analysis, Systematic review

## Abstract

**Background:**

Attention deficit hyperactivity disorder (ADHD) is one of the most common neurodevelopmental disorders of children and adolescents, with a significant impact on health services and the community in terms of economic and social burdens. The objective of this systematic review will be to evaluate the comparative efficacy and safety of pharmacological and non-pharmacological treatments in children and adolescents with ADHD.

**Methods:**

Searches involving PubMed/MEDLINE and the Cochrane Database of Systematic Reviews will be used to identify related systematic reviews and relevant randomized trials. Search results will be supplemented by reports from the regulatory and health technology agencies, clinical trials registers and by data requested from trialists and/or pharmaceutical companies. We will consider studies evaluating pharmacological interventions (e.g. stimulants, non-stimulants, antidepressants), psychological interventions (e.g. behavioural interventions, cognitive training and neurofeedback) and complementary and alternative medicine interventions (e.g. dietary interventions, supplement with fatty acids, vitamins, minerals, aminoacids, herbal treatment, homeopathy, and mind-body interventions including massage, chiropractic, acupuncture, yoga, meditation, Tai chi). Eligible control conditions will be placebo, waitlist, no treatment and usual care. Randomized controlled trials of a minimum of 3 weeks duration will be included. The primary outcomes of interest will be the proportion of patients who responded to treatment and who dropped out of the allocated treatment, respectively. Secondary outcomes will include treatment discontinuation due to adverse events, as well as the occurrences of serious adverse events and specific adverse events (decreased weight, anorexia, insomnia and sleep disturbances, anxiety, syncope and cardiovascular events). Two reviewers will independently screen references identified by the literature search, as well as potentially relevant full-text articles in duplicate. Data will be abstracted and risk of bias will be appraised by two team members independently. Conflicts at all levels of screening and abstraction will be resolved through discussion. Random-effects pairwise meta-analyses and Bayesian network meta-analyses will be conducted where appropriate.

**Discussion:**

This systematic review and network meta-analysis will compare the efficacy and safety of treatments used for ADHD in children and adolescents. The findings will assist patients, clinicians and healthcare providers to make evidence-based decisions regarding treatment selection.

**Systematic review registration:**

PROSPERO CRD42014015008.

## Background

Attention deficit hyperactivity disorder (ADHD) is considered a chronic condition starting in childhood that is comprised of a persistent pattern of symptoms of hyperactivity, impulsiveness and/or lack of attention, which is more frequent and severe than usual for that child’s age, and causing a significant functional impairment in school or work performance and in the activities of daily life [1-5]. ADHD is one of the most common neurodevelopmental disorders of children and adolescents [3,5], with a considerable impact on health services and the community in terms of economic and social burdens [6-9]. The recent Global Burden of Disease Study (GBD 2010) revealed worldwide estimates of 26 million children and adolescents with ADHD, representing 491,500 disability-adjusted life years (DALYs) [7-9].

According to evidence-based guidelines [10-15], the most common recommended treatment options for ADHD include pharmacological and psychological interventions. Stimulant medication is generally recommended as first-line therapy for school-age children and adolescents with severe ADHD, along with implementation of behavioural interventions also recommended [10-15]. In many Western countries, medications currently used for the treatment of ADHD include stimulants (e.g. methylphenidate and amfetamines), non-stimulants (e.g. atomoxetine, clonidine and guanfacine) and sometimes antidepressants. During the past decade, diagnosis rates for ADHD [16,17], medication prescription [11,18-20] and use of complementary and alternative medical therapies by young people [21,22] have risen substantially. However, controversies and public debate over the appropriate diagnosis and treatment of ADHD continue to exist [23-28].

Despite the extensive body of research into the epidemiology, pathophysiology and treatment of ADHD [2,3,5,29], less emphasis has been placed on methodologically sound comparative research questions evaluating and comparing different ADHD treatment options [24]. For example, which ADHD treatment modality works best in children and adolescents, psychological or pharmacological interventions? Among broad groups of treatment interventions, is there any particular medication or psychotherapy which is clinically superior (or inferior) to others? Is there a unique role of complementary and alternative medicine used in the treatment of children and adolescents with ADHD? Which treatment comparisons of ADHD interventions is there sufficient data and for which comparisons are more trials required?

Given the clinical and scientific relevance of these questions, several important systematic reviews have been published in the literature. Some of these reviews are outdated [30-32]. Some reviews were focused on one particular treatment approach only [33-40], most often medications; while others compared medications and psychological interventions [41,42] for ADHD without synthesizing all outcome measures of clinical importance. To our knowledge, none of these previous reviews attempted to establish evidence-based hierarchies for the efficacy and safety of all pharmacological and non-pharmacological treatments in a comprehensive review using network meta-analysis. Network meta-analysis is a relatively new evidence synthesis approach which allows the synthesis of data from both direct (head-to-head, when treatments are compared within a randomized trial) and indirect comparisons (when treatments are compared between trials by combining results using a common comparator) [43,44].

The aim of this systematic review is to address the following research question: For children and adolescents with ADHD, what is the comparative efficacy and safety of competing pharmacological and non-pharmacological treatments?

## Methods

The proposed systematic review will be conducted in accordance with the reporting guidance provided in the Preferred Reporting Items for Systematic Reviews and Meta-analyses (PRISMA) statement (http://www.prisma-statement.org/).

This systematic review and network meta-analysis protocol is registered in the PROSPERO database (CRD42014015008.).

### Study eligibility criteria

#### Type of studies

Only randomized controlled trials (RCTs) of a minimum of a 3-week duration will be included in this review (3 weeks per treatment arm in parallel-group studies and 3 weeks in the first randomisation period for crossover studies). This duration has been chosen because existing research suggests that 3 to 4 weeks on stable medication is the minimum length of treatment chosen in trials designed to measure dose responses [45] or treatment efficacy [32] in subjects with ADHD.

RCTs comparing one pharmacological treatment or non-pharmacological treatments (as monotherapy or in combination) against another or against placebo/control in the treatment of children and adolescents with ADHD will be included.

For trials which have a crossover design, only data from the first randomisation period will be considered due to concerns over carryover effects [46].

Studies of both first-line treatment (defined as the first intervention given to patients) and second-line treatment (defined as the second intervention administered either as a result of failure of or suboptimal response to first-line treatment) will be included. We are interested in studies reporting results of at least one of the outcomes of interest as well as the number of ADHD patients enrolled in each treatment arm and the number of ADHD patients with events in each treatment arm.

#### Type of participants

Studies that enrolled children and adolescents (under 18 years of age) with a clinical diagnosis of ADHD as per either the Diagnostic and Statistical Manual of Mental Disorders (e.g. DSM-IV criteria) or the International Classification of Diseases (e.g. ICD-10 hyperkinetic disorders) criteria will be sought. All ADHD subtypes (e.g. combined type, predominantly inattentive and predominantly hyperactive/impulsive) will be considered for inclusion.

Studies including patients with comorbid conditions (such as anxiety, depression, epilepsy or other medical conditions) will also be eligible for inclusion.

#### Type of interventions

We will consider studies evaluating the following treatments:

### Pharmacological interventions

Pharmacological interventions refer to the treatment of ADHD using medication, under the supervision of a medical professional. Studies evaluating any of the following drugs at any therapeutic dose will be considered:

- Stimulant drugs: methylphenidate, dexmethylphenidate, dexamfetamine/dextroamfetamine, mixed amphetamine salts, lisdexamfetamine.
- Non-stimulant drugs: atomoxetine, guanfacine, clonidine.
- Other approved or unapproved drugs used in ADHD: stimulants (modafinil) and antidepressants (bupropion, venlafaxine, reboxetine, desipramine, imipramine, nortriptyline, clomipramine, amitriptyline).

### Psychological interventions

A diverse range of psychological therapies is available for the treatment of ADHD in children and adolescents. Based on a previous systematic review [47], psychological interventions will be categorized into three domains.

- Behavioural interventions: those interventions directed at changing behaviours (increasing desired behaviours and decreasing undesired behaviours), based on social learning principles and other cognitive theories. These include classical contingency management, behaviour therapy (mainly through mediators such as parents or teachers) and cognitive behaviour therapy (such as verbal self instruction, problem solving strategies or social skills training [47]). These treatments are usually offered in several sessions over time, either through training the mediator(s) or the child or both.
- Cognitive training: working memory training incorporating adaptive schedules that are hypothesized to strengthen ADHD-deficient neuropsychological processes. As in Sonuga-Barke et al. (2013) [47], we will only retain studies including training interventions that aim to directly train a cognitive function, or working memory, or attention.
- Neurofeedback using the visualization of brain activity to teach children to increase attention and impulse control. Neurofeedback is commonly based on electroencephalography; sensors are placed on the scalp to measure activity, and measurements displayed using video displays or sound. By learning to control their brain activity based on behavioural principles of operant conditioning, it is hypothesized that ADHD patients will learn to regulate the associated attentional states and processes [47].

*A priori*, the duration of the psychological interventions will have to be between time frames of ADHD drug therapy (e.g. at least 3 to 12 weeks for initial short-term phase of treatment, at least 24 weeks for mid-term treatment and more than 48 weeks for long-term treatment).

### Complementary and alternative medicine

Complementary and alternative medicine (CAM), as defined by the National Center for Complementary and Alternative Medicine (NCCAM) - U.S. National Institutes of Health (http://nccam.nih.gov/), is a group of diverse medical and health care systems, practices and products that are not presently considered to be part of conventional medicine. Complementary medicine includes treatments that are used together with conventional medicine, whereas alternative medicine treatments are used in place of conventional medicine. A diverse range of CAM interventions are being used for the treatment of ADHD in children and adolescents. Based on NCCAM and expert [48,49] taxonomy, we will categorize CAM into three different types:

- Dietary interventions, such as:
  i. Restricted elimination diet or ‘few foods approach’ (exclusion of items associated with food hypersensitivity, sometimes referred to as an oligoantigenic diet).
  ii. Artificial food colour elimination from child’s diet (e.g. removing food colours such as azo dyes, tartrazine, carmoisine, sunset yellow, brilliant blue, indigotine, allura red, quinoline yellow or ponceau 4R).
  iii. Any other food related interventions.
  - Supplementary interventions:
  i. Polyunsaturated fatty acids (PUFA e.g. omega-3 and omega-6 fatty acids).
  ii. Vitamins (e.g. vitamin B6, vitamin B9, vitamin B12, vitamin C, multiple vitamins).
  iii. Minerals (e.g. magnesium, zinc, iron, calcium).
  iv. Aminoacids (e.g. acetyl-L-carnitine, gamma-aminobutyric acid, glycine, L-tyrosine).
  v. Herbal treatment (e.g. Ginkgo Biloba, Ginseng, St John’s Wort/*Hypericum perforatum*, Valerian).
  vi. Homeopathic treatment.
  vii. Any other supplementary interventions.
  - Mind- and body-based interventions:
  i. Massage, chiropractic and osteopathic manipulation, acupuncture, yoga, meditation, Tai chi, etc.

Control comparators. Eligible control conditions will be placebo (psychological or pill), waitlist (in psychological studies), no treatment or usual care. These may act as vital links for the incorporation of indirect evidence in the treatment networks, and are thus important to include (see data synthesis subsection).

#### Types of outcome measures

The pre-specified primary endpoints will be pragmatic outcomes such as the proportion of patients who responded to or dropped out of the allocated treatment.

- Treatment efficacy (as a dichotomous outcome). We will use response rate as a dichotomous outcome instead of a continuous ADHD symptom score to make the interpretation of results easier for clinicians, patients, parents, teachers, caregivers and decision makers. We will use the number of patients who respond to treatment, based on improvements on standardized rating scales used in clinical trials such as the overall ADHD Rating Scale (ADHD-RS) [50], the Clinical Global Impression-Improvement scale (GGI-I) [50], the Clinical Global Impressions-Severity scale (CGI-S) [51], the Swanson, Nolan and Pelham (SNAP) Rating Scale [52], the Conner’s Rating Scale [53] or any other validated rating scale, at the end of treatment. Many studies define response to therapy as 25% to 30% or greater improvement in core symptoms (e.g. ADHD-RS ≥25% to 30%), a global rating of ‘much’ or ‘very much’ improved (e.g. CGI-I ≤2) or ‘no symptoms’ or ‘minimal symptoms’ (e.g. CGI-S ≤2) [54]. In this review, any definition of response to therapy and any version of validated scales with pre-defined cut-off points for this specific age group will be accepted. We will capture the response criteria used in each trial and data will be extracted in order to explore potential impact on treatment effects if there are different cut-off points used for the same scale, or exclusion of those studies not using established cut-off values (e.g. other than ADHD-RS ≥25% to 30% or CGI-I ≤2). If the original authors report several outcomes corresponding with our definition of treatment response, we will give preferences to the following scales in this order: 1) CGI, 2) ADHD-RS, 3) SNAP, 4) Conner’s and 5) other. This approach was based on expert opinions from our review team. We will also give preference to measures rated by clinicians (followed by teachers/parents and patients). Responders to treatment will be calculated on an intention-to treat basis based on the total number of randomly assigned participants, irrespective of how original study investigators reported data. We will contact authors for missing outcome data or unclear information (e.g. up to three times). Where the number of responders to treatment is unreported and contact with authors fails to acquire this data, we will use an approach applied in recent research [55] to impute outcomes for the missing participants assuming that they did not respond to treatment. When dichotomous outcomes are not reported in studies, but baseline scores, endpoint means and standard deviations (SD) of the rating scales are provided, we will attempt to estimate the number of patients responding to treatment with the validated imputation method previously employed by Furukawa et al. (2005) [56].
- All-cause treatment discontinuation (as a dichotomous outcome), defined as the proportion of patients who leave the study early for any reason as defined by the authors at the longest available follow-up, will also be collected.

Secondary outcomes will include the following:

- Tolerability of treatment (as a dichotomous outcome) measured by the proportion of patients who have left the study early due to adverse events as defined by the authors at the longest available follow-up.
- Serious adverse events (as a dichotomous outcome) defined as the occurrence of any untoward medical event that results in death, is life-threatening, requires inpatient hospitalization or prolongs existing hospitalization, results in persistent or significant disability/incapacity, is a congenital abnormality/birth defect, or is an Important Medical Event. Important Medical Events are events that may have been considered as serious adverse events when, based upon medical judgement, they may jeopardize the patient and may require medical or surgical intervention to prevent one of the outcomes listed above.
- Specific adverse events (as dichotomous outcomes) including the occurrence of decreased appetite, decreased weight, anorexia, insomnia and sleep disturbances, anxiety, syncope, any cardiovascular effect (e.g. hypertension, alterations in heart rate). Study-specific definitions of these events will be recorded to account for variations in definition.

Because of the chronic course of ADHD, we will differentiate outcomes measured during initial short-term treatment (e.g. the first 6 weeks of treatment with a range 3 to 12 weeks), mid-term treatment (e.g. 24 weeks of treatment with a range 13 to 48 weeks) and long-term treatment (e.g. more than 48 weeks). This arbitrary distinction does not imply that more than 24 weeks of treatment defines optimal length of treatment but follows current recommendations on the clinical investigation of pharmacological treatments for ADHD [45].

### Search methods for identification of studies

Based on our awareness of a large number of existing reviews and meta-analyses that can be utilized, an unlimited primary search for RCTs will not be conducted. In its place, we will use a staged approach to study identification, beginning with the identification of relevant randomized trials included in systematic reviews searched for in PubMed/MEDLINE and the Cochrane Database of Systematic Reviews (publication years 2005 and onward), existing systematic reviews, meta-analyses and health technology assessment (HTA) reports which we are aware of [30-42,57-62]. A draft search strategy is included in the Appendix. From identified systematic reviews, we will screen reference lists of both included and excluded studies.

Next, we will search PubMed/MEDLINE to identify other additional relevant RCTs published outside the time frames of these reviews. We will compile a list of the unique PubMed/MEDLINE identification numbers of all relevant articles from the systematic review search and perform a related articles search. This technique has been shown to be effective in identifying relevant studies, has been used recently by reviews in several clinical fields and increases efficiency in study identification in the presence of an already large evidence base [63,64]. These searches will be supplemented by searches in alternative databases (PsycINFO, CINAHL and AMED [65]), and by scrutiny of clinical trial registers (including www.clinicaltrials.gov), HTA agencies (e.g. the National Institute for Health and Care Excellence in the UK, the U.S. Agency for Healthcare Research and Quality and the Canadian Agency for Drugs and Technologies in Health), regulatory bodies (e.g. the U.S. Foods and Drug Administration and the European Medicines Agency) and review of references of relevant papers and clinical practice guidelines.

We will also contact authors of primary publications, collaborators and/or sponsors of clinical trials to check if they are aware of any trials we may have missed.

### Data collection and risk of bias assessment

Eligible RCTs identified from our searching efforts will be screened by two researchers and will be verified by a third researcher of the team to confirm whether each study meets our eligibility criteria. Using a pre-designed form that will be piloted initially on a small number of included studies, the same reviewers will be also responsible for extraction and verification of data on general characteristics (e.g. average age, gender distribution, duration of ADHD, initial severity of ADHD, patient comorbidity history, past/present medication use, mean or median follow-up), characteristics related interventions (such as drug dose or therapist competence in the case of psychotherapy interventions, e.g. therapist qualification, years of experience), outcomes and study design from included studies. If outcomes are not reported at the predefined time points, we will extract data as close as possible to that time point.

The Cochrane Risk of Bias tool [66], which considers sequence generation, allocation concealment, and blinding and other aspects of bias will be used to assess the study risk of bias. The overall rating of risk of bias for each study will be the lowest rating for any of the criteria (e.g. if any domain is scored high risk of bias, the study will be considered high risk of bias). Any discrepancies between reviewers for any of the above steps will be discussed by the reviewers until consensus is achieved.

We will also contact authors of primary publications, collaborators and/or sponsors of clinical trials for missing outcome data or unclear information.

### Data synthesis

We will begin with a narrative overview of studies included in the review which will provide insights regarding descriptive characteristics of the study populations (e.g. age, gender, ADHD subtype, comorbidities, severity of illness) and trial characteristics, describing the types of comparisons made as well as other important variables (such as year of publication, geographic region and sponsorship in case of drug trials). We will use GRADE methodology [67] for evaluating the quality of evidence for outcomes (e.g. high quality, moderate quality and low quality).

#### Standard pairwise meta-analysis

We will perform standard pairwise meta-analyses using a random effects model when data are available. We will evaluate heterogeneity by estimating the variance between studies (chi-square test [68] and *I*^2^ statistic [69]). We will report the results as odds ratios (ORs) and corresponding 95% confidence intervals (CIs). Studies of first-line treatment (defined as the first intervention given to patients) and second-line treatment (defined as the second intervention administered either as a result of failure of or suboptimal response to first-line treatment) will be analysed separately due to the heterogeneity of the intervention.

#### Network geometry

We will describe and present graphically the geometry of the treatment network of comparisons across studies to determine if a network meta-analysis is feasible; we will also identify parts of the treatment networks with considerable evidence versus little or no evidence [70].

The geometry of the treatment network (or network geometry) addresses what the shape of the treatment comparisons looks like in terms of the number of included interventions (e.g. treatment nodes), the extent to which there are trials comparing different pairs of these interventions (e.g. the adjoining lines or ‘edges’) and the numbers of patients associated with different comparisons. By studying and presenting the network geometry, one can develop an understanding of how strong the evidence is for some treatment comparisons and whether specific comparisons are selected, under-represented or even avoided in identified studies [70,71].

Depending on the pattern of studies included, we will explore whether sensitivity analyses related to network geometry are feasible using different classifications to see how stable our results are. We will consider issues including the consideration of treatment nodes for different drug dosages or conducting analyses including all behavioural interventions as a class versus independent analyses (e.g. parent training only, parent/child training or parent/child/teacher training). Control comparators will initially be treated as separate nodes in the networks and combined if effect sizes are relatively similar for them.

#### Network meta-analysis

If the assumptions of homogeneity and similarity are judged reasonable based on review of study characteristics within and across connections in the network of interventions, network meta-analysis will be carried out for each clinical outcome separately. Network meta-analysis is a statistical method used to synthesize information from a network of trials addressing the same question but involving multiple treatments, as well as situations involving the availability of both direct and indirect data for comparisons of interest [43,44,71,72]. Network meta-analyses have been applied in many fields of medicine and clinical psychiatry [55,73,74] to evaluate jointly the comparative safety and effectiveness of multiple available interventions for a condition of interest, even when some of them have not been directly compared in primary research studies. This evidence synthesis method uses both direct (head-to-head) and indirect evidence from all RCTs to be combined into a single effect size for each pairwise comparison and can also be used to estimate comparisons between pairs of treatments which have not been compared in individual studies. By including the combination of direct and indirect evidence, a network meta-analysis may increase precision in estimates and facilitate simultaneous comparisons while within-trial randomization is preserved [43].

In our review, network meta-analyses will be performed within a Bayesian framework using WinBUGS 1.4.3 (MRC Biostatistics Unit, Cambridge, UK) with random effects models adjusting for correlation of multi-arm trials [72] using vague prior distributions throughout. We will report the results as median ORs and corresponding 95% credibility intervals (CrIs), which are the Bayesian analogue of 95% CIs. In addition to estimating ORs, we will also estimate median treatment ranks with 95% CrIs as well as the Surface Under the Cumulative RAnking curve (SUCRA) values [75]. SUCRA values are expressed as percentages; if a treatment is certainly the best, its SUCRA value would be 100%, and if a treatment is certainly the worst, its SUCRA value would be 0%. Forest plots of summary effect sizes as well as rankograms [75] will be used to present main findings.

We will assess model convergence on the basis of inspection of Brooks-Gelman-Rubin plots [76] and Monte Carlo errors.

#### Exploring sources of heterogeneity

Findings from risk of bias assessments of included studies will be used to inform sensitivity analyses including meta-regression [77] or exclusion of higher risk of bias studies and/or small studies to address the impact of perceived study deficiencies. Meta-regression and/or removal of studies from the treatment network to address clinically important variations between studies such as mean age, gender, ADHD severity, presence of comorbidities and year of publication (as a proxy measure of potential changes in clinical practice over time) will also be explored.

If the data permits, we will apply separate subgroup analyses by age-group (e.g. children: <12 years old vs adolescents: 12 to 17 years old).

We will also conduct sensitivity analyses according to the following variables: study design (e.g. including only studies where both the assessor and the patient were blind, sometimes referred as ‘double-blind’ trials), imputation (including only studies without imputation of response rates), response rate definition (e.g. using the most common definition in the included studies) and rating informant (e.g. including only clinician ratings).

#### Assessment of inconsistency

When performing a network meta-analysis, we rely on the assumption of consistency (equivalency of treatment effects from direct and indirect evidence) across the different comparisons in the network. The consistency of results will be examined by comparing the results obtained via pairwise meta-analysis versus network meta-analysis. This will also be examined by fitting both consistency and inconsistency models for network meta-analysis and comparing the deviance information criteria (DIC) between models [78], with smaller values indicative of better fit and a difference of 5 or more being considered as important. If both models have a similar fit to the data as indicated by their DIC values, we will conclude there does not appear to be evidence of inconsistency. Scatterplots of the residuals from both models will also be examined.

## Discussion

This systematic review will evaluate the comparative efficacy and safety of pharmacological and non-pharmacological interventions used in children and adolescents with ADHD. In addition, this review could provide hierarchical information of the multiple competing interventions for ADHD in terms of comparative efficacy (response rates) and safety (all-treatment discontinuations, discontinuations due to adverse events, serious adverse events and selected adverse events). This will produce clinically relevant information that may facilitate understanding the benefit-risk profiles of pharmacological interventions, psychological treatments and CAM in ADHD.

There are several strengths and limitations of our planned methods. In terms of strengths, we will comprehensively review a significant amount of data from both published and unpublished evidence from a range of sources (e.g. journal articles, clinical study reports, trialists/sponsors communications). Also, we have selected for our primary efficacy outcome of response rate to use a dichotomous rather than continuous measure (e.g. standardized mean difference), because from a clinical perspective, syntheses of continuous outcomes measured on different scales can be difficult to interpret [56,79]. However, a limitation of this method is that there can be a substantial information loss when continuous outcome variables are dichotomized [80]. Another limitation is that based on knowledge from previous reviews, we anticipate identifying studies using different study designs, diverse durations, small sample sizes and different end-point definitions, which may increase statistical heterogeneity. Further, the possibility of selective outcome reporting bias (mainly, for unpublished data on harms) in ADHD trials could be a potential limitation of this review.

We hope that our findings will assist patients, clinicians and healthcare providers to make evidence-based decisions regarding treatment selection. Our findings could also potentially be useful for informing the development of evidence-based guidelines as well as the future design of a research agenda of new randomized trials in the field.

## Appendix

Appendix: Key terms for PubMed/MEDLINE search.

Medical condition terms:

#1. “(attention deficit disorder with hyperactivity OR adhd OR hyperkinetic* OR inattent* OR impulsivity OR hyperkinesis OR tdah)”

Population group terms:

#2. “(child OR children OR adolescent* OR pediatric* OR paediatric*)”

Systematic review/meta-analysis terms:

#3. “(meta-analy* OR metanaly* OR metaanaly* OR met analy* OR integrative research OR research integration OR research overview* OR collaborative review* OR systematic review* OR systematic overview* OR evidence-based review* OR evidence-based OR overview* OR meta-review* OR review of reviews OR technology assessment* OR HTA)”

Interventions terms:

#4. “(methylphenidate OR equasym OR ritalin OR concerta OR rubifen OR tranquilyn OR phenidylate OR methyl phenidate OR dexmethylphenidate OR focalin OR metilfenidato OR dextroamphetamine OR dexamphetamine OR dexamfetamine OR elvanse OR vyvanse OR venvanse OR tyvense OR d amphetamine OR amphetamine OR Adderall OR dexedrine OR lisdexamfetamine OR atomoxetine OR tomoxetine OR strattera OR guanfacine OR guanfacina OR tenex OR intuniv OR clonidine OR kapvay OR nexiclon OR catapres OR modafinil OR bupropion OR venlafaxine OR reboxetine OR desipramine OR imipramine OR nortriptyline OR clomipramine OR amitriptyline OR antidepressant* OR contingency management OR management techniques OR contingency techniques OR psychosocial interventions OR psychosocial treatment OR psychosocial therapy OR social skills training OR social skills intervention OR social skills treatment OR problem solving intervention OR problem solving treatment OR problem solving training OR problem solving therapy OR behavior modification OR cognitive behavior treatment OR cognitive behavior therapy OR cognitive behavior training OR parent training OR parent counselling OR parent support OR school-based OR classroombased OR school intervention OR classroom intervention OR teacher training OR after-school or remedial teaching OR peer tutoring OR computer assistance learning OR task modification OR curriculum modification OR classroom management OR education intervention OR multimodal intervention OR multimodal treatment OR multimodal therapy OR multimodal intervention OR multimodal treatment OR multimodal therapy OR educational intervention OR and verbal self-instruction training OR cognitive training OR attention training OR working memory training OR cognitive remediation OR executive function training OR and cognitive control OR neurofeedback OR EEG biofeedback OR neurotherapy OR slow cortical potentials OR few foods diet OR elimination diet OR oligoantigenic diet OR restriction diet OR food intolerance OR food allergy OR and food hypersensitivity OR food color OR food dye OR Feingold diet OR Kaiser Permanente diet OR K-P diet OR tartrazine OR azo dye OR carmoisine OR sunset yellow OR brilliant blue OR indigotine OR allura red OR quinoline yellow OR ponceau 4R OR essential fatty acid OR long-chain polyunsaturated fatty acids OR PUFA OR omega-3 OR omega-6 OR docosahexaenoic acid OR eicosapentaenoic acid OR arachidonic acid OR vitamin B6 OR pyridoxine OR vitamin B9 OR folate OR vitamin B12 OR cobalamin OR magnesium OR zinc OR iron OR calcium OR aminoacid OR amino acid OR carnitine OR L-carnitine OR L-tyrosine OR tyrosine OR tryptophan OR glycine OR melatonin OR taurine OR 5-HTP OR phenilalananine OR aspartame OR ginkgo OR ginkgo biloba OR ginseng OR St John’s Wort OR hypericum perforatum OR rhodiola OR chamomile OR valerian OR bacopa OR pinus marinus OR massage OR chiropractic OR osteopathic manipulation OR acupuncture OR yoga OR meditation OR Tai chi OR complementary medicine OR alternative medicine OR complementary and alternative medicine)”

Combination of terms:

#5. #1 AND #2 AND #3 AND #4

## Abbreviations

ADHD: attention deficit hyperactivity disorders
ADHD-RS: ADHD Rating Scale
CAM: complementary and alternative medicine
CGI-I: Clinical Global Impression-Improvement scale
CGI-S: Clinical Global Impressions-Severity scale
DALYs: disability-adjusted life years
DIC: deviance information criteria
DSM: Diagnostic and Statistical Manual of Mental Disorders
HTA: health technology assessment
ICD: International Classification of Diseases
NCCAM: National Center for Complementary and Alternative Medicine
RCTs: randomized controlled trials
SNAP: Swanson, Nolan and Pelham rating scale
SUCRA: Surface Under the Cumulative RAnking curve
